# 

**Published:** 1857-09

**Authors:** 


*An Associate Editor.*—The appearance of a new name upon our cover for this month may call for an explanation. The "Senior Editor" has found himself many times overburdened with the labor incident to the entire care of both the editorial and business interests of the Journal, and he gladly accepts the assistance of his friend whose name is announced on the title-page. It is believed that this addition to the editorial force will secure a greater variety and interest in our pages, and in the promptness of its issue.

---

*Thanks.*—A paragraph, hinting toward dunning, in our last number, has drawn out many cash replies, accompanied in the majority of instances with remarks so complimentary to the Journal, that the remittance seemed doubled in value. Our friends have our thanks.

